# The diagnostic–clinical chasm: Work in progress?

**DOI:** 10.4102/ajlm.v5i1.586

**Published:** 2016-10-31

**Authors:** John N. Nkengasong

**Affiliations:** 1 International Laboratory Branch, Division of HIV & TB, US Centers for Disease Control and Prevention, Atlanta, Georgia, United States

The *African Journal of Laboratory Medicine* (AJLM) has completed another exciting year by publishing a series of high impact articles that address key aspects of disease prevention and management. The driving forces that underpin the fundamental values of a journal are the felt impact the journal has on the practice of the discipline, in this case laboratory medicine in Africa, and how it guides and helps shape the future of the profession. The series of papers published in this year’s issue satisfy these requirements.

The articles published in AJLM in 2016 fall into four broad categories of diagnostics: availability, access, uptake, and impact on patient care or disease surveillance. With regard to access, several papers address various aspects of the quality of diagnostics and ways to improve the critical interface between laboratorians and clinicians. For example, Jegede and colleagues describe the completeness of laboratory request forms submitted by clinicians in Nigeria.^1^ This is an area that needs considerable work, as clinicians cannot be provided with accurate and timely diagnostic test results for effective management of patients, if laboratory test request forms are not completed accurately. Related to this, Mahomed and colleagues show that management of laboratory expenditures in primary care clinics in South Africa resulted in significant cost savings.^2^ This is vital information, as cost poses a considerable barrier to access to quality diagnostic tests.

More solutions are needed and the chasm between availability of diagnostics and clinicians’ use of the test results will continue to be a work in progress. Will the availability of innovative approaches for diagnostics narrow the chasm? AJLM looks forward in 2017, albeit with anxiety, to how the acceleration of access to diagnostics in Africa through the use of modern approaches could be a game changer in addressing this chasm and impacting public health practice in Africa. For instance, as precision medicine – the ability to individualise patient care with a combination of information unique to an individual to identify prevention and treatment strategies based on genetic, environmental and lifestyle factors – continues to take centre stage in the 21st century in the developed world, laboratory diagnostic testing will play an even greater role. However, the availability of laboratory testing to meet the demands of precision medicine in Africa in the 21st century will require disruptive innovation processes to bridge the diagnostic–clinical chasm. This is reminiscent of how mobile phones became game changers in Africa, making communications affordable and easy.

Similarly, the new generation of non-Sanger-based sequencing technologies, also called next generation sequencing, are becoming a game changer in DNA sequencing due to their extraordinary speed, thus facilitating remarkable achievements and applications, and may also contribute to narrowing the diagnostic–clinical chasm. In developed countries, the increasing availability and affordability of next-generation sequencing technologies is rapidly changing the practice of microbiology. Next-generation sequencing has the potential to revolutionise the practice of public health in Africa by rapidly and accurately providing in-depth details on outbreak-causing pathogens, and decreasing dependence on more time consuming and expensive conventional diagnostic techniques. The question is how could such technologies be effectively established in public health laboratories in Africa to better prepare them for outbreak investigations and other applications? For instance, how would next-generation sequencing technologies add value to Babalola Salu and colleagues’^3^ work on identifying imported Zaire Ebola virus disease from Liberia into Nigeria and subsequent contact tracing, or the rapid molecular confirmation of Lassa fever imported into Ghana as reported by Bonney and colleagues?^4^

Bridging the diagnostic-clinical chasm will continue to be a keen area of implementation science research to identify technological and cultural barriers that must be addressed in order to facilitate uptake and use of patient results and disease surveillance in Africa.
